# 270. Secondary bacterial infection in hospitalized COVID-19 patients. Experience in Colombia, South America

**DOI:** 10.1093/ofid/ofac492.348

**Published:** 2022-12-15

**Authors:** Fernando Rosso, Sarita Rodriguez, Isabel L Zapata-Vasquez, Eric Tafur, Diana M Martinez, Tania Guzmán, Daniela Ramírez-Pérez, Isabella Franky-Ortiz

**Affiliations:** Fundación Valle del Lili, Cali, Valle del Cauca, Colombia; Fundación Valle del Lili, Cali, Valle del Cauca, Colombia; Fundacion Valle del Lili, Cali, Valle del Cauca, Colombia; Universidad Icesi, Cali, Valle del Cauca, Colombia; Fundacion Valle del Lili, Cali, Valle del Cauca, Colombia; Fundación Valle del Lili, Cali, Valle del Cauca, Colombia; Universidad Icesi, Cali, Valle del Cauca, Colombia; Universidad Icesi, Cali, Valle del Cauca, Colombia

## Abstract

**Background:**

Secondary infections are common among severe COVID-19 patients, increasing complications and mortality risk. These infections are not well characterized in Latin America and the Caribbean.

**Methods:**

A cross-sectional observational study of adult patients with COVID-19 admitted to the hospital Fundación Valle del Lili in Cali-Colombia from March 2020 to March 2021. Demographic data, clinical characteristics, laboratory parameters, and clinical outcomes were collected. We describe secondary infection, antibiotic therapy, and antibiotic resistance profiles. Secondary infection was defined if the diagnosis occurred ≥48 hours after hospital admission for COVID-19.

**Results:**

A total of 2138 patients with COVID-19 were analyzed; 350 (16.3%) presented secondary infection. 60% were male; the median age was 65 years [IQR: 55-72]. Glucocorticoid therapy was indicated in 335 patients (96.3%). 281 received high doses and 54 low doses. Bacterial infections were the most common, affecting 81.3 % of patients, followed by fungal (14.4%) and viral (4.3%) infections. Most bacterial isolates were orotracheal secretion, blood, urine, and bronchoalveolar lavage fluid culture. The three most frequently identified bacteria were *Klebsiella pneumoniae*, *Staphylococcus aureus*, and *Pseudomonas aeruginosa*. Most of the initial isolates were not antibiotic-resistant (75-89.7%). Empiric antibiotic therapy was indicated in 346 patients (98.9%), 268 received carbapenems (76.6%), 267 Vancomycin (76.3%), and 233 cefepime (66.6%). Of the 350 patients, 327 (93.4%) required management in the intensive care unit, and overall mortality was 35.4% (124/350).

**Conclusion:**

Our results showed a lower frequency of secondary infection than previous reports; However, a high frequency of broad-spectrum antibiotics usage was found despite a high prevalence of non-resistant bacteria. Further studies are needed to establish the best approach for antibiotics therapy.

**Disclosures:**

**All Authors**: No reported disclosures.

